# Linoleic acid exhibits anti-proliferative and anti-invasive activities in endometrial cancer cells and a transgenic model of endometrial cancer

**DOI:** 10.1080/15384047.2024.2325130

**Published:** 2024-03-11

**Authors:** Jianqing Qiu, Ziyi Zhao, Hongyan Suo, Sarah E. Paraghamian, Gabrielle M. Hawkins, Wenchuan Sun, Xin Zhang, Tianran Hao, Beor Deng, Xiaochang Shen, Chunxiao Zhou, Victoria Bae-Jump

**Affiliations:** a Department of Obstetrics and Gynecology, the Second Hospital of Shandong University, Jinan, PR, China; b Division of Gynecologic Oncology, University of North Carolina at Chapel Hill, Chapel Hill, NC, USA; c Department of Gynecologic Oncology, Beijing Obstetrics and Gynecology Hospital, Capital MedicalUniversity, Beijing Maternal and Child Health Care Hospital, Beijing, China; d Lineberger Comprehensive Cancer Center, University of North Carolina at Chapel Hill, Chapel Hill, NC, USA

**Keywords:** Linoleic acid, endometrial cancer, apoptosis, cell cycle, invasion, cellular stress

## Abstract

Emerging evidence has provided considerable insights into the integral function of reprogramming fatty acid metabolism in the carcinogenesis and progression of endometrial cancer. Linoleic acid, an essential fatty acid with the highest consumption in the Western diet regimen, has shown pro-tumorigenic or anti-tumorigenic effects on tumor cell growth and invasion in multiple types of cancer. However, the biological role of linoleic acid in endometrial cancer remains unclear. In the present study, we aimed to investigate the functional impact of linoleic acid on cell proliferation, invasion, and tumor growth in endometrial cancer cells and in a transgenic mouse model of endometrial cancer. The results showed that Linoleic acid significantly inhibited the proliferation of endometrial cancer cells in a dose-dependent manner. The treatment of HEC-1A and KLE cells with linoleic acid effectively increased intracellular reactive oxygen species (ROS) production, decreased mitochondrial membrane potential, caused cell cycle G1 arrest, and induced intrinsic and extrinsic apoptosis pathways. The anti-invasive ability of linoleic acid was found to be associated with the epithelial-mesenchymal transition process in both cell lines, including the decreased expression of N-cadherin, snail, and vimentin. Furthermore, treatment of *Lkb1*^*fl/fl*^*p53*^*fl/fl*^ transgenic mice with linoleic acid for four weeks significantly reduced the growth of endometrial tumors and decreased the expression of VEGF, vimentin, Ki67, and cyclin D1 in tumor tissues. Our findings demonstrate that linoleic acid exhibits anti-proliferative and anti-invasive activities in endometrial cancer cell lines and the *Lkb1*^*fl/fl*^*p53*^*fl/fl*^ mouse model of endometrial cancer, thus providing a pre-clinical basis for future dietary interventions with linoleic acid in endometrial cancer.

## Introduction

Endometrial cancer (EC) is the most common cancer in gynecologic malignancies and the sixth leading cause of cancer-related deaths in women, with an estimated 66,200 new cases and 13,030 deaths in the United States in 2023.^1^ The most substantial risk factor for EC is unopposed endogenous or exogenous estrogen exposure without sufficient opposition by progestin. Obesity, age, race, and genetic predisposition also increase the risk of EC.^2–4^ Although early stage patients with EC tend to have a good prognosis, those who are diagnosed at an advanced stage often encounter the threat of recurrence, followed by a lack of reliable treatment strategies.^5,6^ As a result, managing the risk factors of EC, especially obesity as a preventive and early intervention measure, is vital to improve the prognosis of EC.^3,4,7^

Fatty acids (FAs), a major component of chemically heterogeneous compounds derived from lipids, perform multiple functions in human cells, including storing energy, modifying the physical properties of lipid membranes, and participating in cell signaling processes and pathological states of the cell.^8^ Linoleic acid (LA) is recognized as an essential fatty acid and the most abundant ω-6 polyunsaturated fatty acid (PUFA) in the Western diet.^9,10^ It is important to note that the only source of LA is food intake, which is mostly derived from meat, eggs, vegetable oils, nuts, soybean, safflower, maize oil, seeds, and sunflowers.^9,11^ Given that the increased supply of vegetable oils over the past few decades has led to a substantial increase in the consumption of LA, which accounts for approximately 6% of total dietary energy, the impact of LA on human health has increasingly attracted attention.^11,12^ During the past three decades, there has been a significant increase in the number of studies being conducted to evaluate the role of LA in human health and wellness, and these studies support the beneficial effect of dietary LA on inflammation and cardiovascular diseases.^12–15^ *In vitro* studies have found that LA, as a precursor of prostaglandin E2 or peroxisome proliferator-activated receptor (PPAR), has conflicting effects on tumor cell proliferation depending on the cancer type, with some showing inhibition and others showing stimulation.^16–21^ Epidemiological and animal experimental studies on the effect of LA on tumor growth have also been inconclusive, reporting that LA may be either beneficial or associated with the promotion of tumor growth, depending on the type of cancer.^12,16,22,23^

Despite several studies examining the effect of dietary LA on EC risk, existing data are inadequate for determining a significant association between the risk of EC and LA.^13,24–27^ Recent metabolomics results showed that LA is one of the most essential metabolites in the blood and tissues of EC patients, compared to women without EC, suggesting that LA may be involved in the carcinogenesis and progression of EC.^28–30^ Thus, the purpose of the present study was to evaluate the effects of LA on cell proliferation and tumor growth in EC cell lines and a transgenic mouse model of EC.

## Results

### LA Inhibited cell proliferation in EC cell lines and tumor growth in a transgenic mouse model

**Figure 1.**
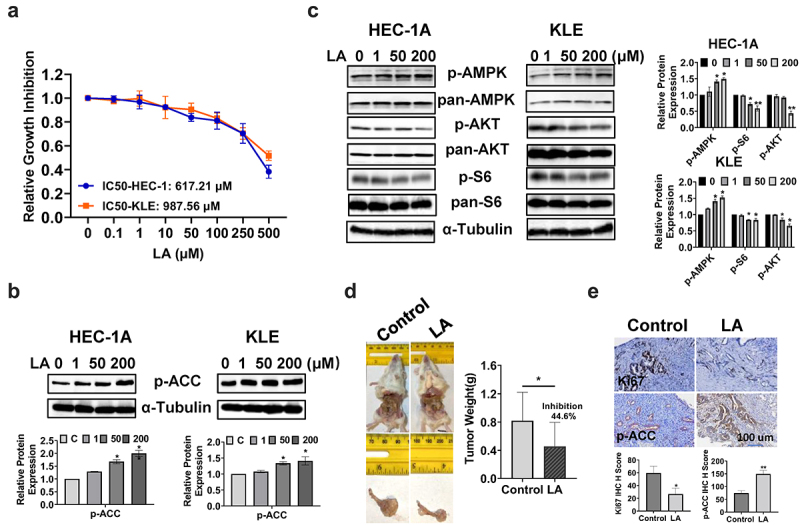
LA Inhibited cell proliferation in EC cell lines and tumor growth in a transgenic mouse model.

The effect of LA on cell proliferation was evaluated in HEC-1A and KLE cells. Both cell lines were treated with different concentrations of LA (0.1–500 μM) for 72 h, and cellular viability was assessed using the MTT assay. The results showed that LA significantly inhibited cell proliferation in a dose-dependent manner in both cell lines. The IC50 doses of LA were 617.21 μM for HEC-1A and 987.56 μM for KLE (Figure 1a). Given that Acetyl-CoA Carboxylase (ACC) is a key player in the biosynthesis and oxidation of fatty acids, the expression of phosphorylated ACC was determined by Western blotting after 24 h of LA treatment in both EC cell lines. LA increased ACC phosphorylation at serine 79 in a dose-dependent manner, reaching a maximum at 200 μM (Figure 1b). To investigate the role of AKT/mTOR and AMPK signaling pathways in cells treated with LA, HEC-1A and KLE cells were treated with 1, 50 and 200 μM LA for 24 h, and the expression of p-AMPK, p-AKT, and p-S6 was detected by Western blotting. The results showed that after LA treatment, the expression of phosphorylated AMPK was upregulated, and phosphorylated AKT and S6 were downregulated in both cell lines (Figure 1c), suggesting that the AKT/mTOR and AMPK pathways are associated with LA-induced cell growth inhibition in EC cells.

The HEC-1A and KLE cells were treated with the indicated doses of LA for 72 h. Cell proliferation was detected using an MTT assay. LA inhibited cell growth in a dose-dependent manner in both the cell types (a). The treatment of HEC-1A and KLE cells with LA for 24 h significantly increased the expression of p-ACC (b). The effect of LA on the AMPK and AKT/mTOR pathways in both cell lines was assessed by western blotting. LA treatment significantly increased the expression of p-AMPK and decreased the expression of p-AKT and p-S6 after 24 h of treatment (c). *Lkb1*^*fl/fl*^*p53*^*fl/fl*^ mice were treated with LA (20 mg/kg, oral, daily) or vehicle for four weeks, and the results showed that LA effectively reduced tumor weight compared with control mice (d). IHC results showed that LA treatment reduced the expression of Ki67 and induced the expression of p-ACC in EC tumor tissues from *Lkb1*^*fl/fl*^*p53*^*fl/fl*^ mice (e). *p < .05, **p < .01. 200

To assess the antitumorigenic effect of LA *in vivo*, *Lkb1*^*fl/fl*^ *p53*^*fl/fl*^ transgenic mice were randomly divided into control and treatment groups (15 mice per group). The mice were treated for four weeks with LA (20 mg/kg, daily) or vehicle daily through oral gavage for four weeks. The mice exhibited normal activity during the treatment. Before treatment, the body weights of the control group and treatment group were 29.15 g and 30.21 g respectively, and after treatment, the body weights of the control group and treatment group were 30.22 g and 30.87 g respectively, indicating that LA treatment did not cause any weight changes. Treatment of mice with LA for four weeks resulted in a significant reduction in tumor weight (44.6%) compared to that in control mice (*p* < .05, Figure 1d). Immunohistochemistry (IHC) staining results indicated that LA treatment significantly decreased the expression of Ki67 by 53.1% compared with that in the control group (*p* < .05). Consistent with the western blotting results, LA significantly increased the expression of p-ACC in endometrial tumor tissues (Figure 1e). These results suggest that LA inhibits EC cell viability and tumor growth *in vitro* and *in vivo*.

### LA Induced cell cycle G1 arrest

**Figure 2.**
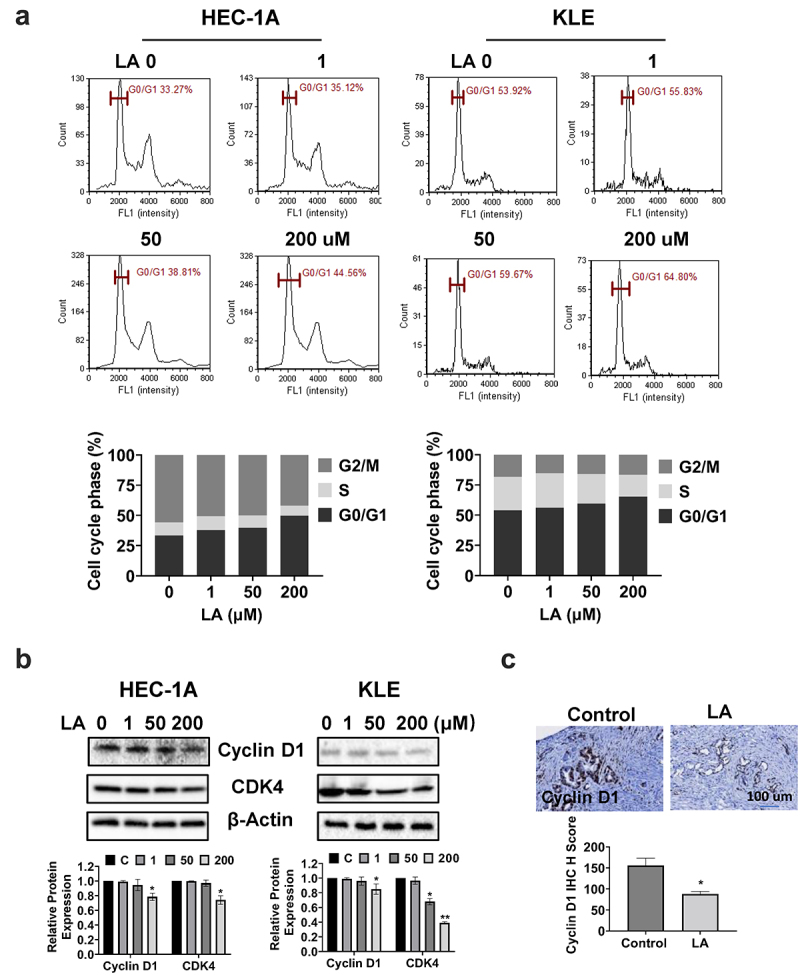
LA Induced cell cycle G1 arrest.

The cell cycle profile was assessed to delineate the underlying mechanism of inhibition of cell proliferation by LA in EC cell lines. After treatment with 1, 50, and 200 µM LA for 36 h, cell cycle profiles of HEC-1A and KLE cells were detected by Image Cytometry. As shown in Figure 2a, LA induced G1 cell cycle arrest in a dose-dependent manner in both the cell lines. The G1 phase arrest population was elongated by 8.26% and 11.17% after treatment with 200 µM LA in HEC-1A and KLE cells, respectively, compared with vehicle-treated cells(*p* < .05). Western blotting showed that LA inhibited the expression of cyclin D1 and CDK4 in a dose-dependent manner in both the EC cell lines after 24 h of treatment (Figure 2b).

HEC-1A and KLE cells were treated with 1, 50, and 200 μM LA for 36 h, and cell cycle progression was analyzed using a Cellometer. LA induced cell cycle G1 arrest in both cell lines (a). Western blotting was performed to detect the expression of cell cycle-related proteins. LA reduced CDK4 and cyclin D1 expression in both cell lines after 24 h of treatment (b). Cyclin D1 expression was measured by IHC in EC tumors from Lkb1^fl/fl^p53^fl/fl^ mice. Treatment of mice with LA for four weeks significantly decreased the expression of cyclin D1 (c) *p < .05, **p < .01.

To examine the effect of LA on the cell cycle *in vivo*, IHC staining was used to detect cyclin D1 expression in EC tumor tissues of *Lkb1*^*fl/fl*^ *p53*^*fl/fl*^ mice treated with LA. The results revealed that LA reduced the expression of cyclin D1 in LA-treated mice by 44.4% compared with that in control mice (Figure 2c). Overall, these results suggest that LA-reduced cell proliferation involves cell cycle G1 arrest in EC.

### LA induced apoptosis

**Figure 3.**
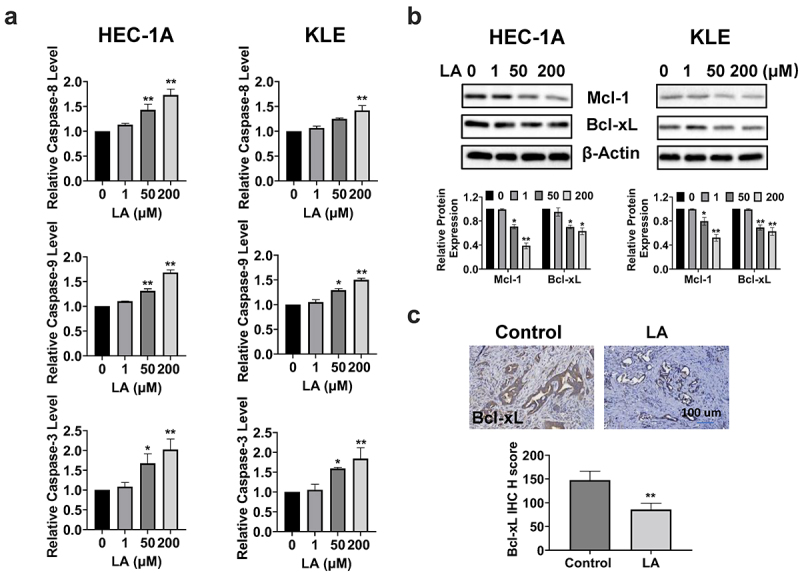
Effect of LA on apoptosis in EC cells and Lkb1^fl/fl^p53^fl/fl^ mice.

To determine whether the inhibition of cell proliferation after LA treatment was associated with apoptosis, we performed cleaved caspase 3, 8, and 9 assays to evaluate the effect of LA on apoptosis in EC cells. After treatment with 1, 50, and 200 µM LA for 16 h, the results indicated that LA significantly increased the activity of cleaved caspase 3, 8, and 9 in a dose-dependent manner in the HEC-1A and KLE cells. LA at a dose of 200 μM increased cleaved caspase 3, 8, and 9 by 2.02-, 1.68, and 1.73 folds, respectively, in HEC-1A cells and by 1.84-, 1.42, and 1.50 folds, respectively, in KLE cells (*p* < .01, Figure 3a). Western blotting results showed that the expression of Bcl-xL and Mcl-1 proteins decreased after LA treatment for 24 h in both cell lines (Figure 3b). Similarly, IHC results confirmed that LA reduced the IHC score of Bcl-xL by 52.2% in LA-treated *Lkb1*^*fl/fl*^ *p53*^*fl/fl*^ mice compared to control mice (Figure 3c). Overall, these results indicate that the inhibition of cell proliferation and tumor growth by LA may be dependent on both extrinsic and intrinsic apoptotic signaling pathways in EC.

Cleaved caspase 3, 8, and 9 activities were determined using ELISA. After treatment with 1, 50, and 200 μM LA for 16 h, the activities of cleaved caspase 3, 8, and 9 were increased in a dose-dependent manner in both HEC-1A and KLE cell lines (a). The expression of Mcl-1 and Bcl-xL decreased in both cell lines after treatment with LA for 24 h (b). LA inhibited the expression of Bcl-xL in EC tumor tissues, as detected by IHC (c).*p < .05, **p < .01.

### LA induced cellular stress

**Figure 4.**
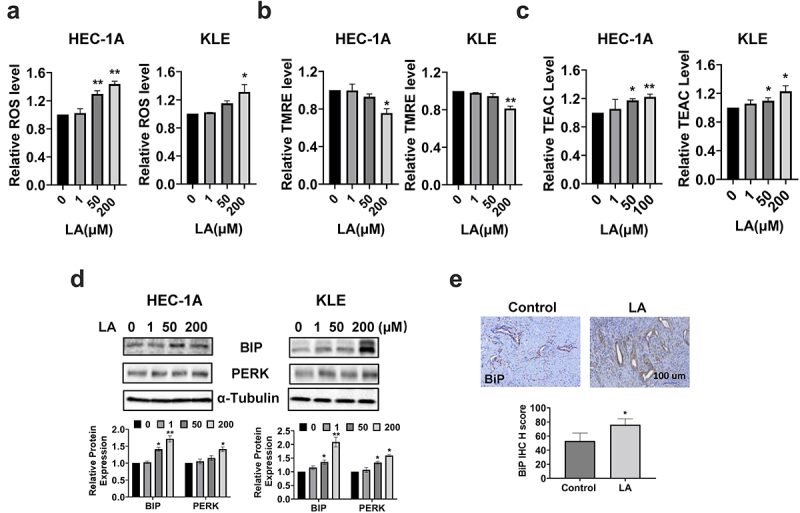
Effect of LA on cellular stress in EC cells and Lkb1^fl/fl^p53^fl/fl^ mice.

To investigate the influence of LA on cellular stress, reactive oxygen species (ROS) levels were measured using the ROS inflorescence indicator, DCFH-DA. After 14 h of treatment with 1, 50, and 200 μM LA, the cellular ROS level increased significantly, especially at 200 µM LA in the HEC-1A and KLE cells. As shown in Figure 4a, ROS levels increased by 38% and 43% with 50 µM and 200 µM LA, respectively, in HEC-1A cells (*p* < .05). In KLE cells, only 200 µM LA significantly increased cellular ROS production (*p* < .05). The potential of the mitochondrial membrane was also assessed using the TMRE assay. Figure 4b shows that treatment of both cell types with 200 μM LA for 14 h effectively reduced the mitochondrial membrane potential by 24.4% in HEC-1A and 18.7% in KLE cells(*p* < .05). The total antioxidant capacity of HEC-1A and KLE cells after LA treatment was determined by TEAC assay. The results showed that LA significantly reduced the antioxidant capacity after treating both cells with 50 and 200 uM LA for 24 hours (Figure 4c). To further characterize the changes in endoplasmic reticulum (ER) stress-related proteins after LA treatment in HEC-1A and KLE cells, the expression of BIP, PERK, and Calnexin was detected by Western blotting after 14 h of treatment. The results showed that LA increased the expression of BiP, PERK, and Calnexin in both cell lines (Figure 4d). The expression of BiP in EC tumor tissues also showed an increasing trend by IHC staining in LA-treated mice compared to that in untreated mice (Figure 4e). These results demonstrate that cellular stress may be one of the mechanisms involved in the inhibitory effects of LA on EC proliferation and tumor growth.

HEC-1A and KLE cells were treated with 1, 50, and 200 µM LA for 14 h. LA significantly increased the ROS levels in both cell lines (a). The TMRE assay showed that 200 µM LA effectively decreased mitochondria membrane potential in HEC-1A and KLE cells (b). The TEAC assay demonstrated that 50 and 200 µM LA significant reduced total antioxidant capacity in both cells after 24 hours of treatment (c). Western blotting results revealed that LA increased the expression of BiP and PERK proteins after treatment with LA for 14 h (d). IHC staining showed an increase in the expression of BiP in LA-treated Lkb1^fl/fl^p53^fl/fl^ mice compared with that in control mice (e). *p < .05, **p < .01.

### LA Inhibited adhesion and invasion

**Figure 5.**
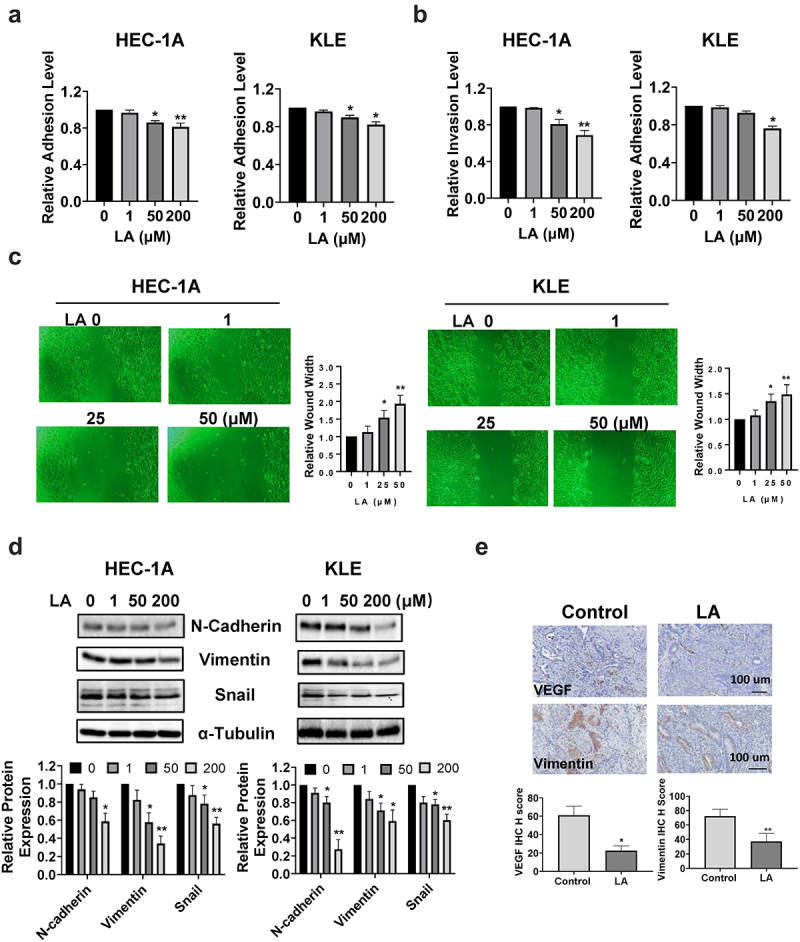
LA Inhibited adhesion and invasion.

The effect of LA on cell adhesion and invasion was investigated using laminin adhesion, transwell, and wound healing assays. The HEC-1A and KLE cells were treated with different concentrations of LA for 2 h in 96-well plates coated with laminin-1. As shown in Figure 5a, cells treated with LA showed significantly decreased adhesion at doses of 50 and 200 μM(*p* < .05). The results of the transwell assay showed that when cells were treated with 200 µM LA for 4 h, the cell invasion of HEC-1A cells and KLE cells was reduced by 31.2% and 24.0%, respectively (*p* < .05, Figure 5b). Similar results were observed in the wound healing assays (Figure 5c). As the concentration of LA increased, the width of the wound gradually increased, indicating that LA inhibited cell migration in both HEC-1A and KLE cells. Next, the role of epithelial-to-mesenchymal transition (EMT) in LA-mediated induction in cell adhesion and invasion was analyzed by Western blotting. HEC-1A and KLE cells were treated with 1, 50, and 200 µM LA for 24 h, and the results demonstrated that 50 and 200 μM LA significantly decreased the expression of EMT-related proteins, including N-Cadherin, vimentin, and snail in both cell lines (Figure 5d). Furthermore, IHC staining showed that LA in the treated mouse group inhibited the expression of VEGF and vimentin in endometrial tumors compared to that in the vehicle-treated mouse group (Figure 5e). Collectively, these results suggest that LA inhibits EC invasion and angiogenesis.

Adhesive and invasive abilities were detected by laminin-1 and transwell assays in EC cell lines, respectively. LA inhibits cell adhesion and invasion in HEC-1A and KLE cells (a,b). The wound healing assay showed that cell migration was inhibited by LA after 48 h of treatment in both cell lines (c). Western blotting showed a descending trend in the expression of EMT-related proteins, including N-Cadherin, vimentin, and snail (d). IHC results indicated that treatment with LA for four weeks in Lkb1^fl/fl^p53^fl/fl^ mice inhibited the expression of VEFG and vimentin in EC tumor tissues compared with that in control mice (e).*p < .05, **p < .01.

## Discussion

LA, the main dietary fatty acid, is a polyunsaturated fatty acid with the highest consumption in the Western diet regimen, with a recommended intake of 12 g/day in healthy women and a plasma concentration of 0.2 to 5.0 mmol/L.^11,31^ Metabolomic profiles in the sera and tumor panels of EC patients are considerably different from those in women without EC, and changes in LA in the sera and tumor tissues of EC patients suggest a functional link between carcinogenesis and EC progression.^28,30^ Thus, our study was performed using physiological plasma concentrations of LA to investigate cell proliferation and tumor growth in EC cell lines and a transgenic mouse model of EC. In the present study, we demonstrated that direct exposure to LA significantly inhibited cell proliferation, induced cell cycle arrest and apoptosis, reduced cell adhesion and invasion, activated AMPK, and suppressed AKT/mTOR signaling pathways in the HEC-1A and KLE EC cell lines. Moreover, treatment with LA for four weeks inhibited tumor growth and reduced the expression of Ki67 and VEGF in the *Lkb1*^*fl/fl*^*p53*^*fl/fl*^ mouse model of endometrioid EC. These results support the concept that LA has anti-tumor effects on EC.

The effects of LA on cell proliferation and tumor growth have been evaluated in various preclinical models. Some studies have shown the pro-tumorigenic effects of LA through prostaglandin E2, insulin/IGF1 receptor, estrogen receptor, and p38 MAP kinase pathways in esophageal and breast cancer cells.^17,19,32^ However, antitumorigenic effects induced by LA have recently been observed in several types of cancer *in vitro* and *in vivo*. LA reduces cell proliferation and induces apoptosis, which is dependent on enhancing the cellular oxidant status and inducing mitochondrial dysfunction in colorectal cancer cells.^18,23^ Activation of the Fas/Fas ligand and caspase pathways by LA has been shown to inhibit the proliferation of gastric adenocarcinoma cells.^20^ The biological basis of the dual functions of LA in promoting or inhibiting tumor cell growth is poorly understood. Tumor type, LA concentration, total dose and duration of exposure, FAS activity, cellular fatty acid composition, and the ratio of LA to other fatty acids are currently considered to be major factors that may affect the function of LA in tumor growth.^33–35^ The current study showed that HEC-1A and KLE cells were susceptible to LA treatment, and both cells exhibited LA-induced ER stress and apoptosis, while IHC results demonstrated increased expression of Bip and decreased expression of Bcl-xL in LA-treated mice compared to the control group. Decreased mitochondrial membrane potential and increased cleaved caspase 9 activity in LA-treated EC cells suggest that exposure to LA leads to mitochondrial dysfunction, thereby activating the mitochondrial apoptotic pathway. These results strongly indicated that the inhibitory effect of LA on cell proliferation and tumor growth was mainly dependent on LA-induced ER stress and apoptosis in EC.

Accumulating evidence suggests a role for reprogramming of fatty acid metabolism in the regulation of cell progression and induction of apoptosis in cancer cells, while cell-cycle regulators contribute to fatty acid metabolism through the regulation of metabolic enzyme.^36–38^ LA promotes cell proliferation and increases the proportion of cells in the S phase of the cell cycle by modulating CDK6, the origin recognition complex, SMAD3, and GADD45 in breast cancer cells.^17^ In contrast, mice injected with the colorectal cancer cell line CT26 and then treated long-term with LA showed cell quiescence, accompanied by an increase in the G0/G1 population and a decrease in the S and G2/M populations, eventually leading to quiescence.^23^ Our data revealed that LA induced cell cycle G1 arrest in HEC-1A and KLE cells and reduced the expression of CDK4 and cyclin D1 in both cell lines, as well as in the EC tumors of *Lkb1*^*fl/fl*^*p53*^*fl/fl*^ mice. Recent studies have demonstrated that the cyclin/CDK4 complex exerts specific effects on lipid and glucose metabolism in addition to regulating cell proliferation.^36,39^ Thus, inhibition of cell cycle regulators, such as CDK4 and cyclin D1, may be another mechanism by which LA inhibits EC cell proliferation and tumor growth.

Fatty acid metabolism is functionally implicated in tumor cell migration, invasion, and metastatic colonization by altering fatty acid uptake and synthesis, modulating metabolic signaling pathways, and controlling epigenetic modifiers and other mechanisms.^38,40^ LA appears to have facilitative or inhibitory functions in regulating the invasive and metastatic cascades in some types of cancer. Treatment with LA effectively promotes an EMT-like process, accompanied by an increase in E-cadherin expression and a reduction in Snail1, Snail2, Twist1, Twist2, and Sip1 expression in MCF10A human mammary epithelial cells.^41^ In triple-negative breast cancer MDAMB-231 cells, LA significantly induced the expression of fascin, which advertised downregulation of breast cancer metastasis suppressor-1 (BRMS1) and upregulation of proteins associated with metastasis, including urokinase-type plasminogen activator, MMP-2, and MMP-9, ultimately resulting in an increased capacity for migration and invasion.^31,42^ Exposure to LA *in vitro* increased the invasive capacity of gastric cancer cells, and increased dietary LA levels stimulated gastric cancer cell invasion and peritoneal metastasis through COX-catalyzed metabolism and ERK activation in a mouse model.^22^ However, weekly intraperitoneal injection of LA significantly reduced the number of metastatic foci in human gastric cancer MKN28 cells and human colon cancer Colo320 cells in the peritoneal cavity of BALB/c nu/nu mice through activation of PPAR γ.^43^ The anti-metastatic potential of LA was similarly obtained in breast cancer MCF-7 cells, where 200 µM LA significantly inhibited 12-O-tetradecanoylphorbol-13-acetate (TPA)-induced MMP-9 expression and cell invasion.^44^ In the present study, adhesion, invasion, and EMT were effectively suppressed by physiological concentrations of LA in EC cell lines, and VEGF expression was inhibited by non-dietary LA administration in EC tissues. These suggests that the suppression of angiogenesis and the EMT process contributes, at least in part, to the inhibition of migration and invasion of EC cells and tumors by LA. Given that EMT-like events are central to carcinogenesis and progression that confer invasive and metastatic properties to EC tumor cells, it is worth investigating the effect of LA on the EMT-like process in our future studies.^45^

In conclusion, our current findings demonstrate for the first time that LA inhibits cell proliferation and tumor growth by inducing apoptosis, cell cycle G1 arrest, activation of AMPK, and inhibition of the AKT/mTOR pathways in EC cell lines and in a transgenic mouse model of EC. Moreover, LA exhibits anti-adhesion and anti-invasion capabilities, accompanied by a significant increase in EMT-like processes and anti-angiogenesis in EC cells and tumors, respectively. Our results identify the biological role of LA in inhibiting tumor growth and invasion of EC, and provide a preclinical rationale for future dietary interventions with LA in EC.

## Materials and methods

### Cell culture and reagents

Two EC cell lines, HEC-1A and KLE, were used. KLE cells were cultured in DMEM/F12 supplemented with 10% fetal bovine serum (FBS), and HEC-1A cells were maintained in McCoy’s 5A medium with 10% FBS. Both media contained 100 U/ml of penicillin and 100 µg/ml of streptomycin. The two cell lines were maintained at 37°C in an incubator with 5% CO_2._ Authentication of these cell lines was performed using the cell bank at the Lineberger Comprehensive Cancer Center at the University of North Carolina at Chapel Hill (UNC-CH). LA was obtained from Sigma-Aldrich (St. Louis, MO, USA). All antibodies were purchased from Cell Signaling Technology (Danvers, MA, USA) and Abclonal Science (Woburn, MA, USA). All the chemicals were purchased from Sigma-Aldrich and Thermo Fisher Scientific (Waltham, MA, USA). During the experiments, we regularly detected mycoplasma contamination in cell cultures every six months using Mycoplasma Detection Assays (Thermo Fisher Scientific).

### Preparation of bovine serum albumin (BSA)-bound LA

One microliter of LA (3.2 M) stock buffer was dissolved in 127 µL of 30% fatty-acid-free BSA (Sigma, A9576) and fully vortexed. The mixture was incubated at 45°C for one hour to obtain a final concentration of 25 mM BSA-bound LA. The mixed solution was filtered through a 0.22-μm filter and stored at − 20°C. The HEC-1A and KLE cells were treated with an equal volume of BSA with different concentrations of LA and an equal volume of BSA solution as a control.

### MTT assay

The MTT assay was used to detect cell viability. HEC-1A and KLE cells were seeded in 96-well plates at a density of 4,000 cells/well and grown for 24 h. Cells were treated the following day with different doses of LA and allowed to grow for an additional 72 h at 37°C. 5 µl of MTT (5 mg/ml) was added to each well for one hour and then replaced with 100 µL of dimethyl sulfoxide (DMSO). The absorbance of each well was measured at 562 nm using a microplate reader (Tecan, Morrisville, NC). The IC50 of each cell line was calculated using an IC50 calculator (ATT Bioquest, Pleasanton, CA, USA). Each assay was repeated at least three times to ensure consistency.

### Cell cycle assay

HEC-1A and KLE cells were cultured in 6-well plates at a concentration of 2.0 × 10^5^ cells/well overnight and then treated with 1, 50, and 200 µM LA for 36 h. The cells were collected using trypsin solution and fixed in 90% methanol for 30 min. RNase A solution (50 μL, 250 µg/ml) was added to the fixed cells for 30 min; the cells were then incubated with propidium iodide (PI) staining solution (2 mg/ml PI, 0.1 mg/ml Azide, and 0.05% Triton X-100) for another 30 min in the dark. The cell cycle progression profile was assessed using a Cellometer (Nexcelom, Lawrence, MA). The distribution of the cell cycle was analyzed using FCS express software (Pasadena, CA). Each experiment was performed at least three times to ensure consistency.

### Cleaved caspase 3, 8, and 9 ELISA assays

HEC-1A and KLE cells were seeded in 6-well plates at a concentration of 2.5 × 10^5^ cells/well for 24 h. The cells were exposed to 1, 50, or 200 µM LA for 16 h. 150–180 ul 1X caspase lysis buffer was added to each well, and the equalized protein value was determined by BCA assay (Thermo Fisher). A reaction buffer containing caspase 3, 8, and 9 substrates (AAT Bioquest), lysis buffer, and an equal amount of protein was added to a black 96-well plate at 37°C for 30 min. The fluorescence intensity was detected using a Tecan microplate reader at different wavelengths of cleaved caspase 3(Ex/Em = 400/505), cleaved caspase 8 (Ex/Em = 376/482), and cleaved caspase 9 (Ex/Em = 341/441). Each experiment was performed three times to ensure consistency.

### Reactive oxygen species (ROS) assay

Alterations in the production of ROS were examined using the Fluorimetric Intracellular Total ROS Activity Assay Kit (AAT Bioquest). HEC-1A and KLE cells (5.0 × 10^3^ cells/well) were seeded in black 96-well plates. After 24 h of growth, cells were treated with different doses of LA for 14 h to induce ROS generation. The cells were incubated with 15 μM DCFH-DA for 30 min, and the fluorescence intensity at Ex/Em 485/530 nm was detected using a Tecan microplate reader. The assay was repeated three times to ensure consistency.

### Mitochondrial membrane potential assay

The potential of the mitochondrial membrane was analyzed using the TMRE assay (AAT Bioquest). HEC-1A and KLE cells were plated in clear 96-well plates and treated with 1, 50, and 200 µM LA for 14 h. The cells were then treated with 800 µM of TMRE solution at 37°C for 30 min. The fluorescence intensity of the TMRE at Ex/Em = 549/575 nm was measured using a Tecan microplate reader. The assay was repeated three times to ensure consistency.

### Trolox equivalent antioxidant capacity (TEAC) assay

The HEC-1A and KLE cells were seeded in 6-well plates(2.5 × 10^5^ cells/ml) overnight and treated with selected doses of LA for 24 hours. After the cells were washed twice with PBS, cells were harvested with cold PBS and sonicated for 10 seconds. The cell lysates were centrifuged at 12,000 rpm for 15 minutes, and 30 µl supernatants with an equal concentration of protein were added to a 96-well plate. The ABTS substrate working solution (120 µl) (Sigma-Aldrich) was immediately added to each well, and the plate was placed on an orbital shaker at room temperature for 5 minutes. A Tecan plate reader was used to read the plate at a wavelength of 410 nm.

### Adhesion assay

A 96-well plate was coated with 100 μl laminin-1 (10 μg/ml) and incubated at 4°C overnight. The plate was rinsed with PBS, and 200 μl blocking buffer was added for 60 min at 37°C. HEC-1A and KLE cells (1.5 × 10^4^ cells/well) treated with 1, 50, and 200 µM LA were seeded into each well, and the plate was incubated at 37°C for 90–120 minutes. Non-adherent cells were aspirated, and 100 μl of 5% glutaraldehyde was added to each well for 30 min. One hundred microliters of 0.1% crystal violet solution were used for staining and 10% acetic acid was used to solubilize the dye. Absorbance was measured at 575 nm using a microplate reader. Each experiment was repeated at least three times

### Wound healing assay

HEC-1A and KLE cells were plated at 6.0 × 10^5^ cells/well in a 6-well plate and incubated overnight. When the cell density reaches 80%, switch to a new medium containing 1% FBS and use a 200 μl pipette tip to form an even wound in each well. The cells were treated with 1, 25, or 50 µM LA for 48 h. Images were taken at 24 and 48 h after scratching. The scratch area was analyzed using ImageJ software (National Institutes of Health, Bethesda, MD, USA). The assay was repeated thrice to assess consistency.

### Transwell assay

The HEC-1A and KLE cells (50,000 cells/well) were starved for 12 h and then seeded into 50 μl of FBS-free medium in the upper chamber of 96-well HTS transwell plate (Life Sciences, Charlotte, NC) coated with 0.5-1X BME (Trevigen, Gaithersburg, MD). The lower chamber contained 150 μl of regular culture medium with different LA. The plate was incubated for 4 h at 37°C and then washed upper and lower chambers with 1X PBS twice. 100 ul Calcein AM solution was added into the lower chamber and incubated at 37°C for30–60 min. The plate was measured at reading fluorescence at EX/EM 485/520 nM in a Tecal plate reader. Each experiment was repeated at least twice for consistency of response.

### Western immunoblotting

HEC-1A and KLE cells were treated with 1, 50, and 200 µM LA for six hours to 30 hours. Cell lysates were prepared using150–180 μl of RIPA buffer (Thermo Fisher). The protein concentration was determined using a BCA protein assay kit (Thermo Fisher). Equal amounts of protein were separated into 10–12% acrylamide gels and transferred onto a PVDF membrane. The membranes were blocked with 5% nonfat dry milk and incubated with the primary antibody (1:1000) overnight at 4°C. The membrane was incubated with the membrane for one hour at room temperature after washing with TBS-T. Immunoblots were developed using enhanced chemiluminescence detection buffer, and bands were visualized using the Bio-Rad Imaging System (Hercules, CA, USA). After development, the membranes were stripped, washed, and re-probed with α-tubulin or β-actin antibodies. Each experiment was repeated at least twice to assess the consistency.

### Lkb1^fl/fl^ p53^fl/fl^ transgenic mouse model of EC

An *Lkb1*^*fl/fl*^ *p53*^*fl/fl*^ transgenic mouse model of EC was used in this study.^46^ The mice were housed at a UNC animal facility on a 12-hour light/12 hours dark cycle. Food and water were provided ad libitum. All mice were handled according to protocols approved by the Institutional Animal Care and Use Committee (IACUC). 5 μl recombinant adenovirus Ad5- CMV-Cre (2.5 ×10^10^ P.F.U, Transfer Vector Core, University of Iowa) was injected into the left uterine horn of mice at the age of six-eight weeks old to induce EC. After 10 weeks of injection, the mice were randomly divided into two groups (15 mice per group) and treated for four weeks with either LA (20 mg/kg, 100 μl per mouse for oral gavage, daily) or vehicle (the control group). All mice were checked daily for any signs of toxicity and weighed weekly during the treatment. The mice were sacrificed at the end of treatment by CO_2_ asphyxiation. EC tumors and blood samples were collected. One-half of the tumor tissues were embedded in paraffin blocks, and the remaining tissue and serum were frozen at −80°C for future use.

### Immunohistochemistry (IHC)

Endometrial tumor slides (4 µm) were incubated with a protein block solution (Dako) for one hour. Primary antibodies (Ki67, Bcl-xL, VEGF, p-ACC, cyclin D1, and BiP) were then added overnight in a cold room. The following day, the slides were rinsed with TBS-T washing buffer three times and then incubated with the appropriate secondary antibodies for one hour at room temperature. The slides were visualized using Signal Stain Boost Immunohistochemical Detection Reagent (Cell Signaling Technology) and scanned using Motic (Feasterville, PA, USA). ImagePro software (Vista, CA, USA) was used to analyze the expression of the target proteins.

## Statistical analysis

Data are presented as mean ± standard error of the mean. The Student’s t-test and one-way ANOVA variance were used in this study. GraphPad Prism 8 statistical software (La Jolla, CA, USA) was used for comparisons. Statistical significance was set at *p* < .05.

## Data Availability

All data generated or analyzed during this study are included in this article. The datasets used and/or analyzed during the current study are available from the corresponding authors upon reasonable request.
